# High frequencies of F1534C and V1016I *kdr* mutations and association with pyrethroid resistance in *Aedes aegypti* from Somgandé (Ouagadougou), Burkina Faso

**DOI:** 10.1186/s41182-018-0134-5

**Published:** 2019-01-04

**Authors:** Aboubacar Sombié, Erisha Saiki, Félix Yaméogo, Tatsuya Sakurai, Takahiro Shirozu, Shinya Fukumoto, Antoine Sanon, David Weetman, Philip J. McCall, Hirotaka Kanuka, Athanase Badolo

**Affiliations:** 1 Laboratoire d’Entomologie Fondamentale et Appliquée, Université Ouaga 1 JKZ, Ouagadougou, Burkina Faso; 2 Laboratory Animal Facilities, The Jikei University School of Medicine, Tokyo, Japan; 3 Center for Medical Entomology, The Jikei University School of Medicine, Tokyo, Japan; 4 National Research Center for Protozoan Diseases, Obihiro University of Agriculture and Veterinary Medicine, Obihiro, Japan; 5 Department of Vector Biology, Liverpool School of Tropical Medicine, Liverpool, UK; 6 Department of Tropical Medicine, The Jikei University School of Medicine, Tokyo, Japan

**Keywords:** *Aedes aegypti*, Insecticide, Resistance, *kdr* mutation, AS-PCR, Sequencing, Burkina Faso, F1534C, V1016I

## Abstract

**Background:**

Resistance to pyrethroid insecticides involving *kdr* mutations is widespread in *Aedes aegypti* (L.) (Diptera: Culicidae) and potentially could impact control efforts in endemic countries. Dengue cases had been sporadic in Burkina Faso for over a decade prior to the 2016–2017 outbreak that resulted in 15,074 suspected cases and 36 deaths, mainly in Ouagadougou. These outbreaks highlighted the lack of information on numerous aspects of the biology, behaviour and insecticide status of local dengue vector populations that are fundamental to vector control.

**Results:**

We investigated the insecticide resistance profiles and the *kdr* mutations involved in pyrethroid resistance of *Ae*. *aegypti* from Somgandé, a district of Ouagadougou. WHO bioassays revealed that the local *Ae*. *aegypti* populations were highly resistant to pyrethroids with mortalities of 15% for permethrin and 37% for deltamethrin. Resistance to carbamates was also detected with mortalities of 55% for propoxur and 90% for bendiocarb, but high mortalities (> 97%) to organophosphates (malathion and fenitrothion) indicated susceptibility. Allele-specific PCR and voltage-gated sodium channel gene sequencing showed a very high frequency (97%) of the F1534C *kdr* allele whilst the V1016I *kdr* mutation frequency was 46%. Association of dual-locus *kdr* mutations was detected for permethrin resistance.

**Conclusion:**

We conclude that in this locality of Burkina Faso, *Ae*. *aegypti* is resistant to pyrethroid and carbamate insecticides but remains susceptible to organophosphates, providing useful information for possible future control.

## Background

Dengue occurs mainly in tropical and sub-tropical areas, especially in urban and semi-urban areas [1], and is a threat to approximately half of the world’s population [2]. In recent decades, dengue cases have increased significantly, largely due to rapidly growing urbanisation that creates vast areas within which the vector *Aedes aegypti* proliferates [3]. Dengue is becoming an acute public health problem [4] in low- and middle-income countries like Burkina Faso which experienced only sporadic cases or rare outbreaks [5, 6] until 2006–2008, when 683 confirmed cases of dengue fever were reported in Ouagadougou in the centre of Burkina Faso and Nouna in the north west [7]. Outbreaks followed with many cases in 2013, in 2016 and again in 2017, when 15,074 suspected cases and 36 deaths occurred mainly in Ouagadougou [8], within which the district of Somgandé has been highlighted as a dengue hotspot since 2013 [9].

Dengue control and prevention rely mainly on vector control, using primarily chemical insecticide classes such as organophosphates and pyrethroids for space spraying and pyrethroid-treated materials for personal protection [10, 11]. Pyrethroid insecticides act on the voltage-gated sodium channel (VGSC) causing depolarization, which disrupts electrical signalling in the nervous system [12, 13] resulting in rapid paralysis and death of mosquitoes known as “knock down”. *Aedes aegypti* has developed resistance to pyrethroids [14] as a result of non-synonymous *kdr* mutations in the VGSC, and via metabolic resistance [15]. To date, only two *kdr* mutations have been found in Africa: V1016I and F1534C [16]. The V1016I mutation in domain II of the VGSC is common in the Americas [15], but has been detected only at very low frequency in Ghana [17]. The F1534C mutation in domain IV in VGSC has a worldwide distribution [15] and was also recently detected in Ghana, but at much higher frequency than V1016I [17]. Metabolic resistance is likely to also have an important role in pyrethroid resistance in *Ae*. *aegypti*, and many genes have been shown to be overexpressed in transcriptomic studies [15]. Some have been demonstrated as being capable of metabolism via in vitro expression studies [18], though quantifying their contribution to resistant phenotypes is difficult and remains to be clarified.

To date, the two *kdr* mutations found in Africa involve nucleotide changes from T to G at codon 1534 resulting in the replacement of phenylalanine by cysteine [19], and from G to A at the codon 1016 resulting in the replacement of valine by isoleucine [20]. These mutants are associated in different ways with pyrethroid resistance in *Ae*. *aegypti*. The F1534C mutation often occurs alone, and its association with resistance to the type I pyrethroid permethrin has been established in vitro [21] and in many field studies [15]. However, association of the V1016I mutation alone with pyrethroid resistance has not been clearly established; in both Mexico and Brazil, co-occurrence of 1016I and 1534C is common and likely driven by selection for resistance [22, 23]. The main objective of this study was to assess the resistance status of *Ae*. *aegypti* to pyrethroids, organophosphates and carbamates and to assess the presence and contribution of *kdr* mutations to pyrethroid resistance in the dengue hotspot of Somgandé district in Ouagadougou (Burkina Faso).

## Materials and methods

### Larval collection

Larvae were collected during the 2016 and 2017 rainy seasons in Somgandé, a district situated in the eastern part of Ouagadougou where relatively high numbers of dengue cases have been recorded since 2013. *Ae*. *aegypti* larvae were collected from artificial outdoor breeding sites such as tires, drums, jars and bowls. Larvae were transported to the laboratory and reared to adults, which were fed with 10% sugar solution. All life stages were maintained in an insectary with 28 ± 1 °C temperature and 68 ± 4% relative humidity.

### Insecticide bioassays

Insecticide bioassays were carried out according to WHO guidelines using the tube susceptibility test kit [24]. Six insecticides were tested: permethrin 0.75%, deltamethrin 0.05%, malathion 5%, fenitrothion 1%, bendiocarb 0.1% and propoxur 0.1%. It should be noted that whilst the diagnostic concentrations for fenitrothion, bendiocarb and propoxur are correct for *Ae*. *aegypti* and that for deltamethrin very close to a provisionally approved concentration, those for malathion and permethrin are higher than the recommended doses [24]. However, in both of these cases, we used the papers applied in the vast majority of other studies of *Aedes* worldwide [15]. Between 80 and 100 unfed 3- to 5-day-old female *Ae*. *aegypti* were exposed to insecticide-impregnated papers for 1 h. Bioassays were carried out at 28 ± 1 °C with 79 ± 7% relative humidity. After the exposure, mosquitoes were transferred to holding tubes and the number of dead mosquitoes was recorded after 24 h. Mortality rates were adjusted with Abbott’s correction when the mortality in the control tubes was between 5 and 20%. The susceptible *Ae*. *aegypti* Liverpool strain was used as control in bioassays to ensure appropriate performance of the tests (i.e. 100% mortality expected in this strain). At the end of the 24-h post-exposure period, live mosquitoes were killed by freezing and stored in silica gel-containing tubes at − 20 °C.

### DNA extraction

Each mosquito was homogenised using a sterilised pestle in 200 μl of a buffer comprising of 0.1 M Tris (pH 9.0), 0.1 M ethylene diamine tetraacetic acid (EDTA), 1% sodium dodecyl sulfate (SDS) and 0.5% diethylpyrocarbonate (DEPC) in a 1.5-ml microtube. The homogenate was incubated at 70 °C for 30 min using a block incubator (ASTEC, B1-516). Following the addition of 44.8 μl of 5 M potassium acetate (KOAc), the mixture was vortexed and kept on ice for 30 min. The mixture was then centrifuged for 15 min at 20,000*g* at 4 °C. A volume of 180 μl of the supernatant was collected in a new 1.5-ml microtube, and 90 μl of isopropanol was added. After vortexing, the mixture was stored at − 25 °C for 30 min and centrifuged at 20,000×*g* at 4 °C for 20 min. The supernatant was discarded, and the DNA pellet was rinsed by adding 200 μl of 70% ethanol. Following centrifugation for 5 min at 20,000×*g*, the pellet was dried at room temperature and dissolved in 20 μl of TE (Tris-EDTA, pH 8.0) buffer.

### Genotyping of F1534C and V1016I mutations

Allele-specific PCR (AS-PCR) followed published protocols for the V1016I (25) and the F1534C (26) mutations in each case with minor modifications.

For detection of the V1016I *kdr* mutation, a reaction volume of 12.5 μl contained 1 μl of target DNA; 2.5 μl of primer mixture comprising of Val1016f (0.25 μM), Iso1016f (0.25 μM) and the Iso1016r common reverse primer (0.125 μM) (Table 1); 2.75 μl of sterile water; and 6.25 μl of Taq polymerase mixture (AmpliTaq Gold® 360 Master Mix, Thermo Fisher Scientific). PCR cycling conditions were initial denaturation for 10 min at 95 °C, 30 cycles of extension at 95 °C for 30 s, 62 °C for 1 min and 72 °C for 45 s, and a final elongation at 72 °C for 5 min.

**Table 1:** List of primer sequences used for detecting allele-specific *kdr* mutations and direct sequencing of the sodium channel regions encompassing these mutations

*kdr* mutations	Primer sequences	References
V1016I	*kdr* genotyping Iso1016f: 5′-GCG GGC ACA AAT TGT TTC CCA CCC GCA CTG A-3′ Val1016f: 5′-GCG GGC AGG GCG GGG GCG GGG CCA CAA ATT GTT TCC CAC CCG CAC CGG-3′ Reverse primer: 5′-GGA TGA ACC GAA ATT GGA CAA AAG C-3′	Martins et al. [25]
	Direct sequencing IIP_F: 5′-GGT GGA ACT TCA CCG ACT TC-3 IIS6_R: 5′-GGA CGC AAT CTG GCT TGT TA-3	Saingamsook et al. [27]
F1534C	*kdr* genotyping M3-F (F1534): 5′-GCG TGA AGA ACG ACC CGA-3′ M3-C (1534C): 5′-GCG TGA AGA ACG ACC CGC′-3′ M3-For: 5′-GGA GAA CTA CAC GTG GGA GAA C-3′ M3-Rev: 5′-CGC CAC TGA AAT TGA GAA TAG C-3′	Li et al. [26]
	Direct sequencing Ge-IIIS6_F: 5′-GCT GTC GCA CGA GAT CAT T-3′ IIIS6_R: 5′-GTT GAA CCC GAT GAA CAA CA-3′	Saingamsook et al. [27]

Genotyping of the F1534C *kdr* mutation required two sets of PCR reactions: the first used a mix of primers M3-For, M3-Rev and M3-F and the second used a mix of M3-For, M3-Rev and M3-C for detection of the 1534F and 1534C alleles, respectively (Table 1). Each PCR reaction volume was 12.5 μl, containing 1 μl of target DNA, 2.5 μl of primer mixture (0.5 μM of final concentration for each primer), 2.75 μl of sterile water and 6.25 μl of Taq polymerase mixture (AmpliTaq Gold® 360 Master Mix, Thermo Fisher Scientific). PCR cycling conditions were initial denaturation for 10 min at 95 °C, 35 cycles of extension at 95 °C for 30 s, 60 °C for 1 min and 72 °C for 30 s, and final elongation at 72 °C for 7 min.

After amplification, the PCR products were mixed with 2.5 μl of 6× Loading Dye Purple Buffer (New England Biolabs), run on either a 3% (V1016I) or a 1.5% (F1534C) agarose gel in TAE buffer (Nacalai, Tesque, Inc., Kyoto, Japan) and stained with ethidium bromide solution for UV visualisation. For the reaction detecting V1016I, the sizes of amplified products were 98 bp for wild-type alleles and 78 bp for mutant alleles [25]. For the reaction detecting F1534C, product sizes for both wild type and mutant alleles were 284 bp [26].

To confirm the results from the allele-specific PCRs, some of the DNA samples were sequenced using primers described by Saingamsook et al. [27] (Table 1) for sequencing the VGSC region. Each PCR reaction mix contained 1 μl of DNA, 0.75 μl of 10 μM forward and reverse primers (IIPF and IIS6_R for V1016I and Ge-IIIS6_F and IIIS6_R for F1534C) (Table 1), 2.5 μl of dNTPs mix (2.5 mM), 1.5 μl of MgSO_4_ (25 mM), 2.5 μl of 10× PCR buffer and 0.5 μl of KOD-plus-neo DNA polymerase (1 U). The mixture was completed up to 25 μl with sterile water. PCR cycling conditions were denaturation at 94 °C for 1 min, 35 cycles of extension at 98 °C for 10 s, 68 °C for 1 min, and a final elongation at 68 °C for 3 min. PCR products were mixed with 5 μl of 6× Loading Dye Purple Buffer (New England Biolabs) and run on a 1.5% agarose gel (Nacalai, Tesque, Inc., Kyoto, Japan). Amplified band of either 581 bp for V1016I or 635 bp for F1534C were cut from the agarose gel for target DNA purification using the QIAEX® II Gel Extraction Kit according to the manufacturer’s protocol.

Sequencing reactions are comprised of 1 μl of purified DNA (5 ng/ul), 0.8 μl of 1 μM of forward (IIP_F or Ge-IIIS6_F) or reverse primer (IIS6_R or IIIS6_R), 2 μl of 5× Sequencing Buffer (Big Dye® Terminator V1.1, V3.1, Applied Biosystems), 0.5 μl of Cycle Sequencing RR-100 (Big Dye® Terminator V3.1, Applied Biosystems) and sterile water for a total reaction volume of 10 μl. Reaction conditions were denaturation for 1 min at 96 °C, 25 cycles of extension at 96 °C for 10 s, 50 °C for 5 s and 60 °C for 4 min. The product was run on an Applied Biosystems 3130x*l* Genetic Analyser, and sequencing chromatograms were analysed using Sequence Scanner 2 (Applied Biosystems).

### Statistical analysis

Susceptibility bioassay results were interpreted according to WHO criteria [24] after 24 h: 98–100% mortality indicates susceptibility, 90–97% mortality suggests suspected resistance and mortality less than 90% indicates resistance. Chi-square tests of independence were performed to compare the mortality rates in 2016 and 2017. *Kdr* allele and genotype frequencies were calculated, and their 95% confidence interval was determined based on Agresti et al. [28]. A chi-square test for deviation from Hardy-Weinberg equilibrium was performed using R with Genetics package version 1.3.8.1. Fisher’s exact test was computed for association between genotype and the resistance/susceptible phenotype using R software version 3.4.3. Cohen’s kappa coefficient (*κ*) was calculated to measure the agreement between the AS-PCR and sequencing methods [29].

## Results

### Insecticide susceptibility

**Fig. 1:**
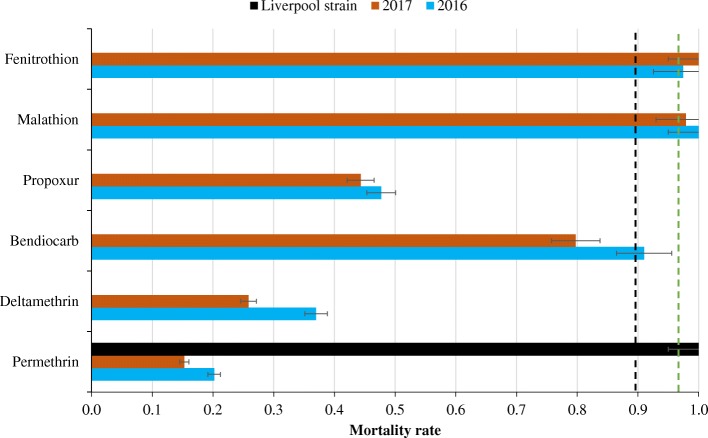
Mortality (with standard errors) of *Ae*. *aegypti* following insecticide exposure in WHO tubes. The green dashed line indicates the threshold for susceptibility, and the black dashed line indicates the threshold for resistance

Bioassay results using the WHO method are summarised in Table 2. Low mortality was observed for pyrethroids with mortalities of 15% in 2016 and 26% in 2017 for permethrin and 20% in 2016 and 36% in 2017 for deltamethrin. Propoxur and bendiocarb were more toxic with mortalities of 44% in 2016 and 55% in 2017 for propoxur and 82% in 2016 and 92% in 2017 for bendiocarb. No difference in susceptibility was observed between 2016 and 2017 for all the insecticides tested (*χ*^2^ < 2, *p* > 0.16). High mortality was observed for the organophosphates fenitrothion and malathion insecticides (mortality between 97 and 100%). Comparatively, the exposure of the Liverpool susceptible *Ae*. *aegypti* strain to permethrin, the less effective insecticide tested, resulted in 100% mortality exposure, showing full susceptibility to this insecticide. These results indicate that the *Ae*. *aegypti* population from Somgandé is susceptible to organophosphates (or very nearly so) but resistant to permethrin, deltamethrin, bendiocarb and propoxur (Fig. 1).

**Table 2:** *Ae. aegypti* mortality rates after exposure to insecticides in 2016 and 2017 using WHO tubes standard bioassay guidelines. The chi-square is calculated, and the probability comparing 2016 and 2017 data and insecticides of the same family are shown

	Mortality (%) with 95% CI								
	Carbamate		*χ*^2^ (*p*)	Organophosphate		*χ*^2^ (*p*)	Pyrethroid		*χ*^2^ (*p*)
	Propoxur	Bendiocarb		Fenitrothion	Malathion		Permethrin	Deltamethrin	
2016	54.74 [44.74–64.37]	91.92 [84.86–95.85]	9.43 (0.002)	97.44 [91.12–99.29]	100 [96.44–100]	0.03 (0.855)	20.19 [13.6–28.9]	36.99 [26.82–48.45]	4.9 (0.02)
2017	44.32 [34.39–54.72]	81.91 [72.93–88.39]	11.19 (0.0008)	100 [96.19–100]	97.87 [92.57–99.41]	0.02 (0.879)	15.28 [8.75–25.32]	25.84 [17.88–35.80]	2.7 (0.09)
*χ*^2^ (*p*)	1.09 (0.29)	0.57 (0.45)		0.03 (0.86)	0.02 (0.88)		0.7 (0.41)	1.98 (0.16)	

Numbers in brackets indicate the 95% CI; *χ*^2^ was obtained by Pearson’s chi-squared test

### Allele and genotype frequencies of the V1016I and F1534C *kdr* mutations

In total, 578 and 554 mosquitoes were genotyped using the allele-specific PCR for the F1534C and V1016I mutations, respectively. At codon 1016, both wild-type (*V*/V) and mutant genotypes (V/I or I/I) were detected, whereas at codon 1534, wild-type genotypes (F/F) were absent. The majority (93%) of mosquitoes were homozygous 1534 C/C, and 21% homozygous 1016 I/I. Allele frequencies were 0.97 (95% CI 0.954–0.975) for 1534C and 0.46 (95% CI 0.429–0.488) for 1016I (Table 3).

**Table 3:** V1016I and F1534C genotype numbers and frequencies in parenthesis and the allelic frequency of the I and C mutations of *Ae*. *aegypti.* The chi-square and the probability comparing the genotypes is given

No. of genotypes and genotype frequency (Frq)								*kdr* allele frequency	
F1534C			HWE *χ*^2^ (*p*)	V1016I			HWE *χ*^2^ (*p*)		
F/F	F/C	C/C		V/V	V/I	I/I		C	I
0 (0.00)	39 (0.07)	539 (0.93)	0.884 (0.347)	163 (0.29)	274 (0.49)	117 (0.21)	0.006 (0.932)	0.97	0.46

Numbers in brackets indicate the genotype frequency

**Fig. 2:**
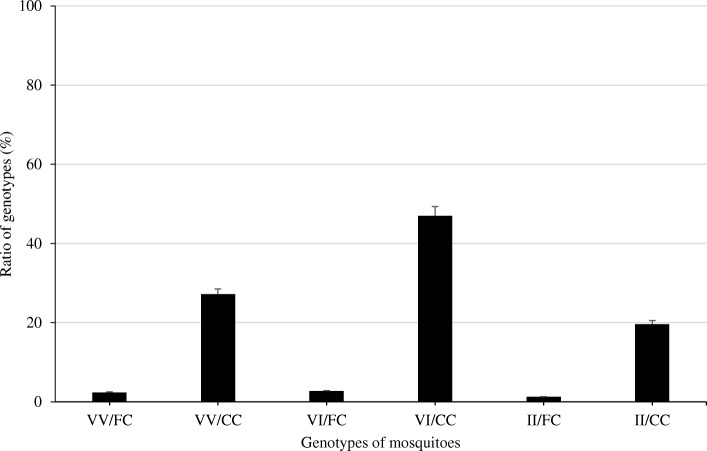
Frequency of the combined V1016I and F1534C genotypes where VV/FC, VV/CC, VI/FC, VI/CC, II/FC and II/CC mean respectively and 1016VV/1534CC, 1016VV/1534CC, 1016VI/1534FC, 1016VI/1534CC, 1016II/1534FC and 1016II/1534CC genotypes

Only six from a possible nine genotypes across the two *kdr* mutations were identified from 552 *Ae*. *aegypti* mosquitoes from which genotypes at both loci were available (VV/FC, VV/CC, VI/FC, VI/CC, II/FC and II/CC) (Fig. 2). The genotype VI/CC was the most frequently observed (47%, *n* = 259), and 20% of individuals were homozygous for both *kdr* mutations (II/CC) (Fig. 2). Overall, the genotype frequency distribution was strongly deviant from Hardy-Weinberg equilibrium (*χ*2 < 1, *p* > 0.34) (Table 3). Forty-eight samples were sequenced for the sodium channel regions encompassing these mutations, and the sequencing data confirmed all six possible genotypes detected by AS-PCR.

### Association of genotypes at V1016I/F1534C with pyrethroid resistance

As shown in Table 4, the frequency of the VV/CC genotype was relatively lower in live (deltamethrin, 11%; permethrin, 16%) than in dead mosquitoes (deltamethrin, 32%; permethrin, 29%). In contrast, the genotype frequency of VI/CC was higher in live mosquitoes (deltamethrin, 61%; permethrin, 52%), and the double mutant genotype II/CC was also detected more frequently in live mosquitoes (deltamethrin, 26%; permethrin, 28%). The association between the dual-locus *kdr* genotypes was significant for permethrin resistance (*p* < 0.001), but not deltamethrin resistance (*p* = 0.116) (Table 4). However, caution is required as the power of the test was limited by the low number of genotypes for dead mosquitoes.

**Table 4:** Co-occurrence of V1016I and F1534C mutations in pyrethroid-exposed mosquitoes. The number of dead and live mosquitoes and their frequencies in permethrin and deltamethrin exposure *Ae*. *aegypti.* The probability (*p* value) of genotype distribution in dead and alive mosquitoes is calculated

	Number of genotypes and their frequencies									*p* value
	VV/FF	VV/FC	VV/CC	VI/FF	VI/FC	VI/CC	II/FF	II/FC	II/CC	
Deltamethrin										
Alive	0 (0.00)	2 (0.03)	7 (0.11)	0 (0.00)	0 (0.00)	40 (0.61)	0 (0.00)	0 (0.00)	17 (0.26)	0.116
Dead	0 (0.00)	0 (0.00)	7 (0.32)	0 (0.00)	0 (0.00)	12 (0.55)	0 (0.00)	0 (0.00)	3 (0.14)	
Permethrin										
Alive	0 (0.00)	0 (0.00)	10 (0.16)	0 (0.00)	2 (0.03)	32 (0.52)	0 (0.00)	0 (0.00)	17 (0.28)	0.00018
Dead	0 (0.00)	2 (0.29)	2 (0.29)	0 (0.00)	2 (0.29)	1 (0.14)	0 (0.00)	0 (0.00)	0 (0.00)	

Numbers in brackets indicate the genotype frequency; mutant alleles are shown in bold

## Discussion

This study investigated insecticide resistance of *Ae*. *aegypti* in Ouagadougou, the capital of Burkina Faso, and indicates that this important arbovirus vector is resistant to pyrethroid and carbamate insecticides but remains susceptible to organophosphates. Although the reasons for these patterns are not known, there are a number of events that may have contributed. Long-lasting insecticidal nets (LLINs) have been promoted by WHO [30] for malaria control throughout Africa with dramatic results in recent years [31]. Scaling up of the distribution of these pyrethroid-treated bednets in Burkina Faso started in 2010 and led to millions of nets being deployed throughout the country. Despite impacts on malaria mortality and morbidity, scaling up probably accelerated the development of pyrethroid resistance in populations of malaria vectors [32] and, given its peridomestic habits, may also have contributed to the development of pyrethroid resistance in *Ae*. *aegypti*. Selection pressure from widespread use of pyrethrum coils and insecticide aerosols in households is also likely [33, 34]. Resistance to carbamates is more difficult to explain because this class of insecticides is not used for vector control in Ouagadougou, though they are used for pest control in urban agriculture in Ouagadougou [35]. In Cameroon, deltamethrin and bendiocarb resistance was recorded in *Ae*. *aegypti* by Kamgang et al. [36] whilst 6 years earlier *Ae*. *aegypti* was found susceptible to deltamethrin and propoxur [37], suggesting a loss of susceptibility to these insecticides with time. However, in our study, no significant change in susceptibility is observed between 2016 and 2017 to all the insecticides tested, a short period indeed.

Resistance profiles from other African countries are quite variable with respect to carbamates, more common in pyrethroids, but remain very rare for organophosphates [16].

Allele-specific PCR genotyping of *Ae*. *aegypti* population from Somgandé revealed the occurrence of V1016I and F1534C mutations, the latter at very high frequency. The allelic frequencies of both mutations are higher than in Ghana, from where these two mutations were first reported in mainland Africa [17]. The near fixation of the F1534C allele in the *Ae*. *aegypti* population of Burkina resulted in an absence of genotypes including only 1016 *kdr* mutations, precluding independent analysis. When analysed together, a significant association with permethrin resistance of dual-*kdr* genotypes was detected in *Ae*. *aegypti* from Somgandé. This is consistent with results from Linss et al. [22] and also the model suggested by Vera-Maloof et al. [38] which suggests a rise in frequency of 1534C followed by a rise of 1016I, with concomitantly increased resistance. No association was detected in our study between *kdr* and deltamethrin resistance, and whilst power was limited because of the relatively small number of dead genotypes available, this was actually even more so for permethrin, suggesting that association between these *kdr* genotypes and deltamethrin might be weaker than for permethrin. However, the lack of any association demonstrated for deltamethrin should be taken with caution, and further work with larger sample sizes is required.

The V1016I *kdr* mutation is often found in association with the 1534C mutation in Southern and Central America [38–40] and in the Caribbean [41]. A similar profile of extreme 1534C frequency and moderate 1016I frequency has been detected in Madeira and is thought to have been imported recently [42]. Further investigation into the origin of the 1016I mutation in Burkina Faso is required as well as investigation of the role of metabolic resistance in *Ae*. *aegypti* [15].

## Conclusion

The study has demonstrated that *Ae*. *aegypti* from a locality of Ougadougou are resistant to pyrethroids and carbamates but susceptible to organophosphates. The V1016I and especially F1534C *kdr* mutations were observed at high frequency and explain at least some of the observed pyrethroid resistance phenotypes. Further work is required to investigate spatial and longer-term temporal variations in the occurrence of these mechanisms and to identify other mechanisms involved in the resistance phenotypes. These results provide important information for the development of effective strategies for dengue control in Ouagadougou.
